# The impact of metformin use on the risk of prostate cancer after prostate biopsy in patients with high grade intraepithelial neoplasia

**DOI:** 10.1590/S1677-5538.IBJU.2017.0046

**Published:** 2018

**Authors:** Lucio Dell'Atti, Andrea B. Galosi

**Affiliations:** 1 Department of Urology, University Hospital “St. Anna”, Ferrara, Italy;; 2 Department of Urology, Marche Polytechnic University, Ancona, Italy

**Keywords:** Diabetes Mellitus, Prostatic Neoplasms, Prostatic Intraepithelial Neoplasia

## Abstract

**Purpose:**

We report our experience on metformin use in diabetic patients and its impact on prostate cancer (PCa) after a high-grade prostatic intraepithelial neoplasia (HGPIN) diagnosis.

**Materials and Methods:**

We retrospectively analyzed 551 patients with a diagnosis of HGPIN without PCa in a first prostate biopsy. The cohort of the study consisted of 456 nondiabetic subjects, and 95 diabetic patients. Among the patients with diabetes 44 were treated with metformin, and 51 with other antidiabetic drugs. A transrectal ultrasound prostate biopsy scheme with 22 cores was carried out 4-6 months after the first diagnosis of HGPIN.

**Results:**

Among 195 (35.4%) patients with cancer, there were statistically significant differences in terms of PCa detection (p<0.001), Gleason score distribution (p<0.001), and number of positive biopsy cores (p<0.002) between metformin users and non-users. Metformin use was associated with a decreased risk of PCa compared with neveruse (p<0.001). Moreover, increasing duration of metformin assumption (≥2 years) was associated with decreasing incidence of PCa and higher Gleason score ≥7 compared with assumption <2 years.

**Conclusions:**

This preliminary experience suggests that metformin use may have some beneficial effects in patients with diabetes and HGPIN; metformin should not be overlooked in these patients because it is neither new nor expensive.

## INTRODUCTION

Prostate cancer (PCa) is the first most common cancer in men worldwide, and the prostate biopsy is the only modality to diagnose this disease (1). Although not diagnostic of PCa on a needle biopsy, many epidemiological, molecular, histopathological, and genetic studies have offered strong evidences that high-grade prostatic intraepithelial neoplasia (HGPIN) is a precursor lesion to development of invasive PCa (2). HGPIN is seen in 4-16% of patients undergoing prostate biopsy (3). Metformin, an oral biguanide, is the first line therapy for many patients with type 2 diabetes (4, 5). Several recent observational studies have shown that metformin can inhibit cancer proliferation, and simultaneously induce cell apoptosis by the AMP-kinase pathway and AMP-kinase-independent mTOR inhibition (6). In this study, we report our experience on metformin use in diabetic patients and its impact on prostate cancer (PCa) after a high-grade prostatic intraepithelial neoplasia (HGPIN) diagnosis.

## MATERIALS AND METHODS

We retrospectively examined 562 consecutive patients underwent transrectal ultrasound prostate biopsy (TRUSBx) by means of a scheme with sampling of 22 prostate regions carried out in our centres from April 2007 to November 2016. Inclusion criteria for this retrospective study with standard 22-core biopsy scheme included a prior diagnosis of HGPIN at the first prostate biopsy. Decision for a first TRUSBx was based upon high prostatic specific antigen (PSA) levels and/or suspicious digital rectal examination (DRE) findings according to EAU (European Association Urology) guidelines. The second biopsy was performed within six months from the first one. For a standardization of the clinical data, patients with a history of surgical treatment for prostatic disease and incomplete clinical data were excluded from our study. Among 562 patients eligible for this study, a total of 551 patients for whom preoperative complete clinical data, use of oral antidiabetic drugs (OADs), and their duration information were available. OADs use and duration information were obtained from patients, medical records and medical database software. The cohort of the study consisted of 456 nondiabetic patients, 44 patients with type 2 diabetes managed with metformin, and 51 diabetic patients managed with other antidiabetic drugs. All patients enrolled in the study signed a consent form for the biopsy procedure. TRUSBx was performed in the operating room under analgesia, and using a ultrasound machine equipped with a 5-9MHz multi-frequency convex probe “end-fire” (GE Logiq 7, Milwaukee, WI, USA). Each transrectal ultrasound performed included an assessment of the volume of the whole prostate, the transition zone, capsular, seminal vesicle characteristics, and a morphological description of potential pathological features. The prostate volume was invariably calculated using prostate ellipse formula (0.52 x length x width x height). After having images of the prostate, sampling was carried out with a 18-Gauge Tru-Cut (Bard Biopsy Systems, Tempe, AZ, USA) needle powered by an automatic spring-loaded biopsy disposable gun. Three experienced urologists of our Department performed a 22-core biopsy scheme, as first intention, including 3 basal samples (2 lateral and 1 medial), 3 parasagittal samples (2 lateral and 1 medial), 2 apical samples (1 lateral and 1 medial), and 3 transitional zone sample on each side. This biopsy scheme was changed based on TRUS findings concerning the size of the prostate and varied from 18 cores from a small prostate to 24 cores for large prostatic glands. The Gleason grading was based on the recommendations of the 2005 International Society of Urological Pathology consensus conference. All histological specimens were analysed internally by our Pathology Department specialized in genitourinary pathology. Cases were not reviewed for the purposes of this study.

### Statistical analysis

Patient age, body mass index (BMI), PSA level, prostate volume (PV), DRE findings, OADs, were analysed as continuous variables and presented with mean and standard deviation. Quantitative variables are presented with absolute and relative frequencies. For the comparison of proportion, the three study groups, chi-square and Fisher's exact tests were used. Student's t-tests were computed for the comparison of mean values. Statistical analyses were performed using Microsoft Excel 2010 platform version 10.1. A p<0.05 was considered to indicate statistical significance.

## RESULTS

**Figure 1:**
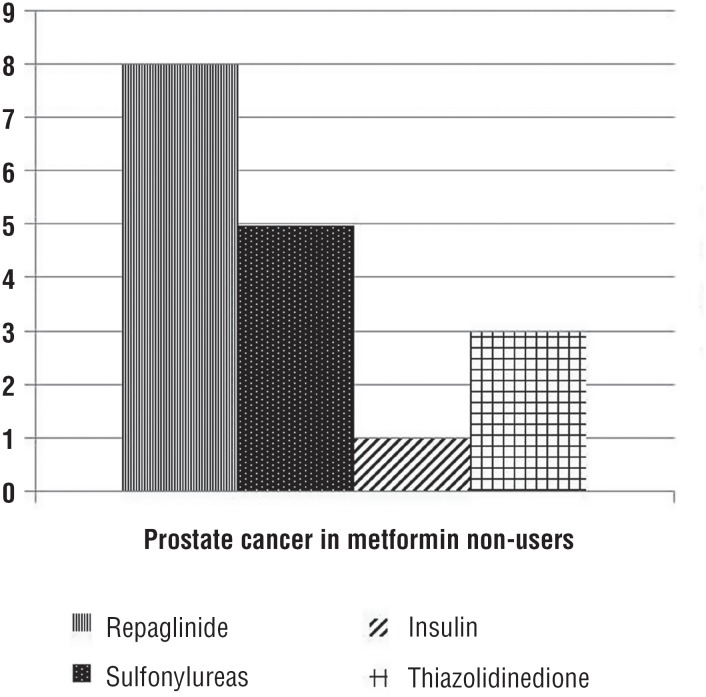
Prostate cancer in patients that do not use metformin.

**Figure 2:**
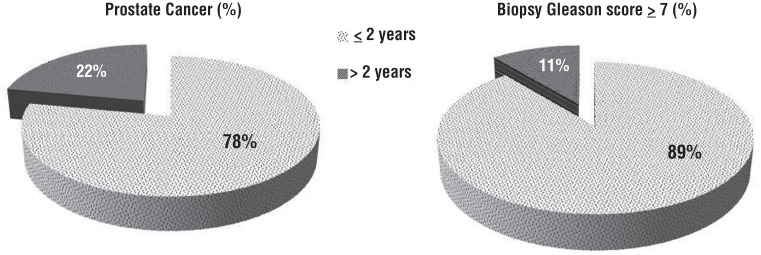
Relation between duration of metformin use, incidence of prostate cancer and Gleason score.

22-TRUSBx scheme was carried out 4-6 months after the first diagnosis of HGPIN. Of 551 patients, 95 (17.2%) were diabetics and 456 were not diabetics. The baseline demographics and clinical characteristics of the 551 patients included in the study are shown in Table-1. Use of other medications for diabetes was fairly common: repaglinide [37.2% (19/51)], sulfonylureas [31.3% (16/51)], insulin [17.7% (9/51)], and thiazolidinedione [13.8% (7/51)]. There were no statistically significant differences between three groups of patients in terms of age, PSA levels, PV, DRE findings. Diabetic patients not treated with metformin were more likely to present a higher BMI (p<0.001), and to have higher Gleason score ≥7 on biopsy (p=0.002). Among 195 (35.4%) patients with cancer, there were statistically significant differences in terms of PCa detection (p<0.001), Gleason score distribution (p<0.001), and number of positive biopsy cores (p<0.002) between metformin users and non-users. The mean duration of metformin use was 5.3±2.7 years. Metformin use was associated with a decreased risk of PCa compared with never-use (p<0.001). However, a reduced risk of PCa was associated with insulin use (Figure-1), but not with other antidiabetic drugs (p<0.002). Moreover, increasing duration of metformin assumption (≥2 years) was associated with decreasing incidence of PCa and higher Gleason score ≥7 compared with assumption <2 years (Figure-2).

**Table 1:** Demographic and clinicopathologic features of patients affected by HGPIN and undergoing transrectal prostate biopsy.

		Non antidiabetic Drug users (n: 456)	Metformin Users (n: 44)	Metformin Non-users (n: 51)	p value
Age (years), mean ± SD		65.1±7.4	64.7±6.9	65.5±7.1	NS
Body Mass Index (kg/m^2^), mean ± SD		26.7±4.6	27.2±3.9	33.8±3.1	<0.001
Prostate volume (mL), mean ± SD		38.4±12.7	36.9±11.3	37.8±12.3	NS
PSA level (ng/mL), mean ± SD		11.3±8.6	10.8±8.8	11.1±8.3	NS
N° biopsy cores, median (range)		21.2 (18-24)	21.7 (18-24)	21.4 (18-24)	NS
**Family history PCa, n(%)**					NS
	No	338(74.1)	34(77.2)	39(76.5)	
	Yes	118(25.9)	10(22.8)	12(23.5)	
DRE, n(%)		181(39.7)	17(38.6)	20(39.2)	NS
Prostate cancer, n(%)		169(37.1)	9(20.5)	17(33.3)	<0.001
Positive biopsy cores, mean ± SD		7.1±2.4	3.4±1.8	6.3±2.2	<0.002
**Cancer laterality, n(%)**					**<0.001**
	Unilateral	112(66.3)	7(77.7)	11(64.7)	
	Bilateral	57(33.7)	2(22.3)	6(35.3)	
**Biopsy Gleason score, n(%)**					**<0.001**
	≤6	103(60.9)	8(88.8)	12(70.5)	
	≥7	66(39.1)	1(11.2)	5(29.5)	

**HGPIN =** High grade prostatic intraepithelial neoplasia; **PCa =** prostate cancer; **DRE =** digital rectal examination; **SD =** standard deviation; **PSA =** prostate-specific antigen; **NS =** not significant.

## DISCUSSION

In diabetic patients, metformin is prescribed as first-line therapy because of optimal tolerability, efficacy in reducing insulin resistance, and low cost (7). Its primary action is the inhibition of hepatic glucose production through an LKB1/AMPK-mediated mechanism, and it also improves insulin sensitivity in peripheral issues (8). However, recent epidemiological studies have shown that metformin can reduce the risk of breast, pancreatic, colon and prostatic cancers and might even improve cancer prognosis (9-11). Metformin has recently collected the interest from the medical community for its potential beneficial effects on PCa outcomes, and its use was significantly associated with increased overall survival and decreased biochemical recurrence (12-15). Moreover, metformin has been demonstrated to inhibit progression in PCa by modifying the expression of cancer suppressor genes and oncogenes in animal and in vitro studies (8). By reducing hyperinsulinemia, metformin can influence multiple other cancer pathways, including IGF (insulin growth factor) and PI3K-AKT/AR signalling, both of which are linked with PCa prognosis and castrate resistance (16). On the contrary, HGPIN is associated with the development of PCa, and patients with HGPIN are more likely to develop cancer than those without HGPIN (36.3% vs. 25%) (17). The clinical importance of recognizing HGPIN is based on its strong association with PCa, so its identification in biopsy specimens warrants further search for concurrent invasive carcinoma. Follow-up biopsy is suggested at 3 to 6 months for 2 years, and thereafter at 12 month intervals for life (18). Nevertheless, no factors seem to be useful in identifying which patients with HGPIN are at risk of PCa progression. Currently, routine treatment is not available for patients who have HGPIN. Prophylactic radical prostatectomy, radiation, and androgen deprivation are not acceptable treatments for patients who have HGPIN only (19). The development and identification of acceptable agents to treat HGPIN would fill a therapeutic void. In a meta-analysis of 9186 men with diabetes and PCa, Stopsack et al. showed that metformin decreased biochemical recurrence and improved overall survival through an antiproliferative effect via inhibition of mTOR (6). There are many studies of the association of metformin with PCa focused on cancer incidence (10, 13-15, 20). Wright et al. (21) reported that among whites with diabetes, metformin resulted in a 44% reduced risk of PCa. However, previous studies have reported conflicting conclusions regarding the impact of metformin on PCa diagnosis (12). One study did not support an association between decreased risk of PCa incidence and use of metformin (22), while Joentausta et al. demonstrated that metformin users had even higher risk of high-grade PCa in men undergoing radical prostatectomy (23). According to our current knowledge, this is the first study that correlates the antidiabetic drugs as a treatment for patients who have HGPIN and risk of PCa progression. Hypothesizing that this relationship might be instituted from the initiation of PCa carcinogenesis we established to perform a preliminary study to analyse whether metformin use might be considered a chemopreventive agent for PCa in those patients with a prior diagnosis of HGPIN. Our data showed that metformin users with a negative re-biopsy after HGPIN diagnosis were proportionately higher than metformin non-users (p<0.001). Moreover, increasing duration of metformin use was associated with decreasing incidence of PCa and cancer-specific characteristics. Preston et al. (24) reported an inverse relationship between PCa risk and duration of metformin therapy. Metformin use <1.5 years was not associated with a risk reduction but durations of >3 years were associated with a decreased PCa incidence. Furthermore, our results suggest that a greater proportion of patients had Gleason scores ≥7 with significant difference between metformin users and non-metformin users in contrast to results reported by various authors (25, 26). A study based on the Canadian population found no association between metformin use and the risk of PCa including Gleason grade. Margel et al. (26) showed the lack of association between metformin use and risk of PCa including Gleason grade. A possible explanation of such discordance might rest on differences of cancer patient population. In our study, prostatic cancers at re-biopsy were found to harbour localized cancer, mostly well or moderately differentiated. However, several limitations need to be acknowledged. A first limitation, we had no data available regarding the ethnic background of the patients. This detail could be of special interest, because in multi-ethnic populations, some subgroups might have more unfavourable PCa characteristics than others. However, Mitin et al. (27) showed that diabetes mellitus was associated with an increased risk of Gleason score 8 to 10, independent of black race. Although we did not expressly documented race, the majority of the patients of our study cohort were white and Italian population. Thus, the number of Asian and black patients was very small and surely did not exceed 1% of the entire cohort. Second, this is a monocentric study with a limited number of antidiabetic users. Third, this retrospective study concerned also patients eligible only for radical prostatectomy; as a consequence older patients (≥74 years old) who are not candidates for surgery, were excluded. This may have influenced the results generalizability and preclude comparative investigation of a potentially risky cancer in metformin users vs. non-users.

## CONCLUSIONS

This preliminary experience suggests that metformin use may have some beneficial effects in patients with diabetes and HGPIN; metformin should not be overlooked in these patients because it is neither new nor expensive. Though our results sustain the chemoprevention effects of metformin on PCa risk among patients with a prior HGPIN, additional studies and randomized clinical trials with more detailed exposure measurement are warranted to evaluate questions about dose and therapy duration.

## ABBREVIATIONS

Pca: = Prostate cancer
HGPIN: = high-grade prostatic intraepithelial neoplasia
TRUSBx: = Transrectal ultrasound prostate biopsy
DRE: = Digital rectal examination
PSA: = Prostate-specific antigen
OADs: = Oral antidiabetic drugs
BMI: = Body mass index
PV: = Prostate volume
